# Management of astigmatism in keratoconus with therapeutic refractive surgery

**DOI:** 10.3389/fmed.2026.1853734

**Published:** 2026-09-10

**Authors:** Hao Zhang, Jorge L. Alió del Barrio, Qingluan Bao, Jorge L. Alió

**Affiliations:** 1 Cornea, Cataract and Refractive Surgery Unit, Vissum (Miranza Group), Alicante, Spain; 2 Division of Ophthalmology, School of Medicine, Universidad Miguel Hernández, Alicante, Spain

**Keywords:** astigmatism, corneal cross-linking, customized photorefractive keratectomy, intracorneal ring segments, keratoconus, toric phakic intraocular lens

## Abstract

Astigmatism is a major determinant of visual impairment in keratoconus (KC), yet in this setting it cannot be understood as a simple cylindrical refractive error. Instead, it reflects a complex optical phenotype generated by anterior and posterior corneal asymmetry, epithelial remodeling, internal optical compensation, and progressive biomechanical instability, often in combination with coma-dominant higher-order aberrations. Beyond reducing monocular image quality, KC-related astigmatism may also compromise binocularity through interocular asymmetry, impaired binocular summation, reduced stereopsis, and visual discomfort. Management therefore depends not only on correcting refractive cylinder, but on distinguishing calculated astigmatic vectors from functionally relevant refractive astigmatism and identifying the relative contributions of irregular corneal optics, including coma, trefoil, and residual low-order error. Measurement remains challenging because corneal and manifest astigmatism frequently diverge, and because results are influenced by centration, analysis zone, device characteristics, and limited repeatability in more advanced disease. Current therapeutic refractive strategies are best viewed within an individualized, phenotype-driven framework rather than a mandatory linear sequence. Corneal collagen cross-linking remains fundamental when progression is present, but has limited refractive predictability. Corneal regularization procedures, including intracorneal ring segments, corneal allogeneic intrastromal ring segments, and customized surface ablation, may be selected when irregular corneal optics are the dominant cause of visual impairment. Once stability and sufficient regularity are achieved, toric phakic intraocular lenses, and in selected cases toric intraocular lenses, can address residual refractive error. Future progress will depend on multimodal imaging, improved phenotyping, and more individualized corneal shape-restoration strategies.

## 1 Introduction

Keratoconus (KC) is a progressive corneal ectasia characterized by stromal thinning and biomechanical weakening, leading to localized corneal steepening, cone-like protrusion, and progressive refractive and optical distortion (1, 2). As the disease advances, the optical phenotype often shifts from predominantly regular astigmatism to a more complex pattern dominated by irregular astigmatism and higher-order aberrations (HOAs), particularly asymmetric third-order aberrations, including vertical coma, horizontal coma, and trefoil (3). Clinically, these changes result in reduced corrected distance visual acuity (CDVA), glare, halos, ghosting, and visual fluctuation that may be disproportionate to the manifest cylinder and not fully correctable with standard refraction (1, 3).

Importantly, astigmatism in KC is not simply a cylindrical refractive error. In this review, refractive astigmatism refers to the manifest cylindrical error measured by subjective or objective refraction; corneal astigmatism refers to astigmatic power derived from corneal topography, including anterior, posterior, or total corneal astigmatism; and irregular astigmatism refers to non-spherocylindrical optical distortion caused by asymmetric corneal shape and HOAs, particularly coma and trefoil. These components are clinically relevant but not interchangeable, and they often diverge in KC (4). Calculated astigmatic vectors should therefore be interpreted as measurement-derived descriptors of corneal or ocular optics rather than direct substitutes for functionally relevant refractive astigmatism, particularly in moderate-to-advanced KC, where visual limitation may be driven more by coma, trefoil, and asymmetric irregular optics than by true cylindrical error (1). This dissociation reflects the asymmetric contribution of the cornea together with the influence of internal optics, including lenticular astigmatism, which may partially compensate for or augment corneal astigmatism (5). In addition, the magnitude and axis of astigmatism may vary over time because of disease progression, epithelial remodeling, contact lens wear, and ocular surface status, further complicating measurement and treatment planning (5).

Beyond monocular optical degradation, KC may also impair binocular visual function. Because the disease is frequently asymmetric, interocular differences in image quality can reduce binocular summation, impair stereopsis, and cause visual discomfort. Accordingly, management of keratoconic astigmatism should aim not only to reduce refractive cylinder, but also to improve overall optical quality and functional vision.

Conventional management is primarily non-surgical and follows a stepwise approach (3). Spectacles may provide adequate vision in mild disease, but their effectiveness declines as irregularity increases. Rigid gas-permeable (RGP), hybrid, and scleral contact lenses remain the mainstay of visual rehabilitation because they mask anterior corneal irregularity by creating a more regular refractive surface (6). However, long-term lens dependence may be limited by intolerance, fitting complexity, ocular surface disease, and lifestyle constraints.

Corneal collagen cross-linking (CXL) is the cornerstone treatment for halting KC progression, but its refractive effect is variable and not sufficiently predictable to serve as a standalone solution for astigmatism correction (7, 8). Once progression is stabilized and corneal shape is sufficiently stable, additional surgical interventions may be considered to regularize the cornea, reduce irregular astigmatism, and correct residual refractive error (1, 9). These include intracorneal ring segments (ICRS) (10), corneal allogeneic intrastromal ring segments (CAIRS) (11), customized excimer laser surface ablation (8), toric phakic intraocular lenses (12) or cataract/refractive lens surgery in selected cases (13).

This review summarizes the current evidence on the surgical management of astigmatism in KC, with a particular focus on evidence-based approaches to its characterization, measurement, and treatment selection.

## 2 Methods

This article was designed as a structured narrative review rather than a systematic review or meta-analysis, aimed at summarizing contemporary evidence on the characterization, measurement, and surgical management of astigmatism in KC. A structured literature search was performed in PubMed to identify English-language articles published between January 2016 and March 2026. The search was last updated on March 31, 2026. The search strategy used combinations of the following terms: keratoconus, astigmatism, irregular astigmatism, corneal topography, corneal tomography, corneal cross-linking, intracorneal ring segments, corneal allogeneic intrastromal ring segments, photorefractive keratectomy, topography-guided, wavefront-guided, phakic intraocular lens, and toric intraocular lens. Articles published before January 2016 were generally excluded unless considered essential to provide historical or technical context.

Evidence was appraised according to the Oxford Centre for Evidence-Based Medicine Levels of Evidence framework (14). In this context, preference was given to systematic reviews and meta-analyses, randomized or comparative clinical studies, and prospective or well-conducted cohort studies. For emerging topics in which higher-level evidence remained limited, selected lower-level studies were considered only when they contributed clinically important and directly relevant information. The level of evidence was used to grade the main clinical recommendations rather than to generate quantitative study weights (Table 1). Practical selection criteria and decision-making suggestions presented elsewhere in this review integrate the available evidence with expert clinical interpretation and should not be interpreted as formal guideline recommendations.

**Table 1:** Oxford CEBM-based grading of main clinical recommendations.

Clinical recommendation	Main evidence base	Supporting references	Oxford CEBM level	Recommendation strength
Progressive keratoconus should be stabilized before refractive optimization, most commonly with CXL.	Systematic reviews, contemporary reviews, and comparative/cohort evidence supporting CXL for progression control.	(3, 7–9)	Level 1–2	Strong
Customized PRK + CXL may be considered in selected mild-to-moderate keratoconus with sufficient stromal reserve and adequate topographic/wavefront data quality.	Meta-analysis of nonrandomized studies, prospective comparative cohorts, and representative clinical series.	(1, 8, 15–29)	Level 2	Moderate
ICRS may be considered in contact-lens-intolerant eyes or eyes with poor spectacle function when cone morphology, corneal clarity, and stromal thickness are suitable.	Systematic review/meta-analysis, indication-focused review, and observational cohort studies.	(10, 30–45)	Level 2–3	Moderate
CAIRS may be considered an emerging corneal regularization option, but current evidence remains heterogeneous and less standardized.	Systematic reviews based mainly on retrospective or prospective case series, technical reports, and short-term observational cohorts.	(11, 46–72)	Level 3–4	Weak-to-moderate
Toric pIOLs may be considered in stable keratoconus with reproducible regular refractive astigmatism and adequate anterior segment anatomy.	Systematic review/meta-analysis and observational cohorts of iris-fixated and posterior chamber toric pIOLs.	(12, 73–106)	Level 2–3	Moderate
Toric IOLs may be considered in cataract patients with stable mild-to-moderate keratoconus and sufficiently regular astigmatism.	Contemporary review, IOL-calculation evidence, and systematic review/meta-analysis of toric IOL implantation in keratoconus.	(13, 107–110)	Level 3	Weak-to-moderate

The Oxford CEBM level reflects the hierarchy of the principal supporting evidence. Recommendation strength represents a qualitative synthesis of the overall evidence base and should not be interpreted as a formal GRADE assessment.

CEBM, Centre for Evidence-Based Medicine; KC, keratoconus; CXL, corneal collagen cross-linking; PRK, photorefractive keratectomy; ICRS, intracorneal ring segments; CAIRS, corneal allogeneic intrastromal ring segments; pIOL, phakic intraocular lens; IOL, intraocular lens.

**Figure 1:**
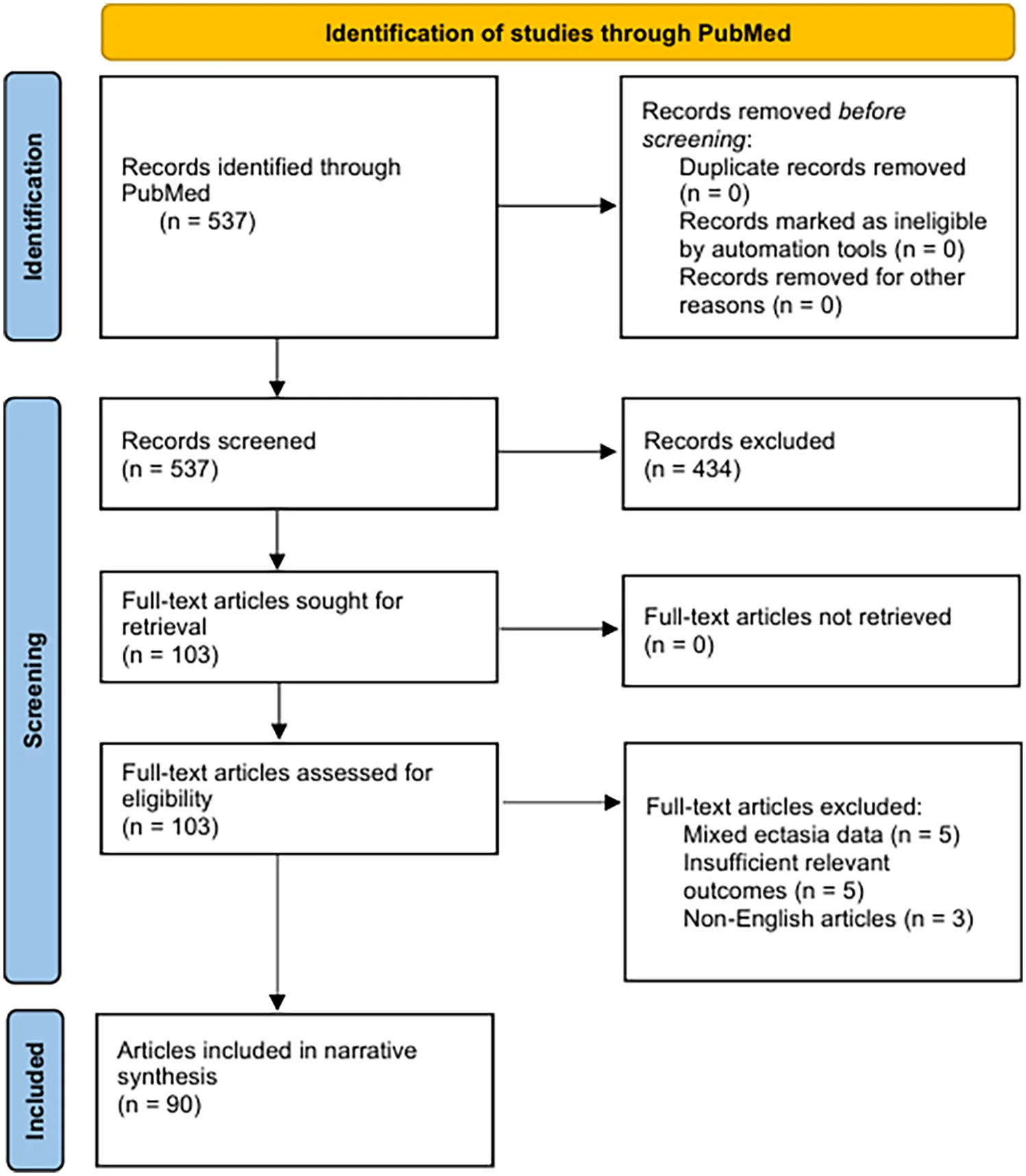
Flow diagram of literature identification and selection for this structured narrative review.

The initial screening was performed by one reviewer (H.Z.), and the selected records were subsequently reviewed by a second reviewer (J.L.A.B.). Any disagreements were resolved by a third senior reviewer (J.L.A.). A total of 537 records were identified through PubMed. After removal of duplicate records and publications that were not directly relevant to the scope of the review, and after full-text evaluation according to the scope and evidence requirements of the review, 90 articles were selected for narrative synthesis (Figure 1). Because this was a structured narrative review rather than a formal systematic review or meta-analysis, no quantitative pooling was performed. When multiple publications or meta-analyses appeared to include overlapping cohorts, particularly in the CAIRS literature, the most recent, comprehensive, or methodologically relevant source was prioritized, and overlapping evidence was not interpreted as independent confirmation. These studies were used to support a structured discussion of current concepts, practical measurement considerations, and contemporary treatment strategies for keratoconic astigmatism.

## 3 Considerations in the preoperative evaluation of astigmatism in KC

### 3.1 Characteristics of astigmatism in KC

Across studies using Scheimpflug tomography (Pentacam), KC shows a large increase in anterior corneal astigmatism (ACA) accompanied by a non-negligible posterior corneal astigmatism (PCA). In the large KC cohort by Naderan et al., mean ACA was 4.16, 4.20, 5.49, and 5.17 D across Amsler–Krumeich stages 1–4, respectively, while mean PCA was 0.85, 0.86, 1.09, and 0.96 D, respectively, supporting the clinical relevance of PCA across disease stages (111). A separate staged KC cohort further suggested that the relative contribution of PCA may be proportionally higher in early KC (112). Vector-based analysis further confirms that posterior astigmatic vectors [e.g., posterior astigmatic polar value (APV)] rise markedly in overt KC, whereas forme fruste KC (fellow eyes) may overlap with normal controls when analysis is restricted to the central 3 mm-diameter zone—highlighting that posterior-surface metrics alone may be insufficient for detecting the earliest disease, depending on the measurement region (113).

Beyond magnitude, KC displays distinctive axis and coupling behavior between anterior and posterior surfaces, but reported agreement varies with definitions and measurement settings. In one large study, ACA was most commonly classified as against-the-rule (ATR), while PCA most commonly showed with-the-rule (WTR) predominance when posterior WTR/ATR classification is adjusted for the negative refractive power of the posterior surface; moreover, anterior–posterior categorical agreement was low (very small kappa), underscoring frequent axis discordance at the individual-eye level despite strong magnitude correlation (111). Another staged analysis similarly demonstrated strong ACA–PCA magnitude correlation that weakens in advanced disease and a tendency for oblique patterns to decrease with progression, suggesting that later stages may evolve toward more “dominant” meridional patterns (112). In contrast, Shajari et al. reported that, in KC eyes, corneal astigmatic alignment became increasingly “vertical” with disease progression, while normal eyes showed a largely vertical posterior axis regardless of anterior alignment (114). Taken together, these findings suggest that KC often increases anterior–posterior coupling, but the measured degree of coupling depends heavily on how axis categories are defined (WTR/ATR vs. vertical/horizontal), whether posterior-axis definitions are inverted, and how the measurement zone is centered.

Critically, KC astigmatism is also spatially heterogeneous and measurement-dependent. In inferotemporal cone KC, changing centration (pupil-centered vs. apex-centered) and zone diameter (3 vs. 6 mm) significantly alters corneal astigmatic vectors; even within paracentral regions, both magnitude and direction vary markedly—effects that are minimal in normal corneas (115). Therefore, when characterizing “KC astigmatism,” it is essential to report (and ideally standardize) the analysis zone and centration, because different settings can yield meaningfully different astigmatic phenotypes and may partly explain discrepancies across studies.

### 3.2 Measuring astigmatism in KC: accuracy, repeatability, and practical pitfalls

Accurate astigmatism assessment in KC requires moving beyond “a single cylinder and axis” toward a measurement strategy that matches the clinical goal (progression monitoring, corneal regularization planning, or lens-based correction). Two concepts are pivotal. First, the clinically experienced cylinder (“manifest” or ocular astigmatism) may diverge from corneal metrics because KC introduces spatially heterogeneous optics and internal compensation (4). Second, even within corneal measurements, the choice of algorithm (anterior-only vs. total cornea), centration, and analysis diameter can change both magnitude and axis, sometimes by clinically meaningful amounts.

What to measure: anterior vs. total corneal astigmatism. Traditional simulated keratometry (SimK) reflects primarily the anterior surface with a standardized keratometric index, which can be problematic in ectatic corneas. This index, commonly *n* = 1.3375, is based on an assumed anterior-to-posterior corneal curvature relationship derived from the Gullstrand model; because KC disrupts this anterior–posterior ratio, anterior-only SimK may misestimate total corneal astigmatism (116). Studies using rotating Scheimpflug imaging demonstrate that total corneal refractive metrics (e.g., true net power or ray-traced total corneal refractive power, TCRP) can differ systematically from SimK in KC, and that total corneal astigmatism varies with measurement configuration (115, 117). Consequently, for refractive decision-making—especially when toric correction is contemplated—reporting total corneal astigmatism (rather than anterior-only astigmatism) is favored as a more complete measurement approach, but it remains not yet validated as producing superior postoperative visual quality in KC.

Centration and analysis zone: a major source of “method-dependent variability.” In KC, the corneal apex/vertex and pupil center are often decentered relative to each other, and the cone is typically off-axis. For example, changing from pupil-centered to apex-centered analysis within a 3 mm zone produced differences of approximately 0.36 D in total corneal astigmatism and 0.48 D in anterior astigmatism, with larger effects when the analysis diameter was expanded or the cone was more decentered (115, 117, 118). These data support a practical message for both clinics and research: centration and zone definitions must be standardized and explicitly reported, because they can alter the inferred astigmatism phenotype.

Repeatability: prioritize repeatability limits over the intraclass correlation coefficient (ICC) when following individuals. Many studies report excellent reliability with high ICCs, but ICC can be misleading in KC cohorts because large between-eye variability may keep ICC values high despite clinically meaningful within-eye measurement noise. Within-device repeatability limits, or the coefficient of repeatability (CoR), are more clinically actionable than ICC, whereas limits of agreement (LoA) should be reserved for between-device or between-method agreement. In a single-center Pentacam HR analysis using five repeated scans, the reported values represented 95% within-device repeatability limits, which were sizeable for key parameters, including Kmax (−1.1D) and thinnest pachymetry (−14 µm), and were particularly poor for astigmatism axis, raising concern about using single measurements to infer progression or to drive axis-dependent nomograms (117, 118). A larger multicenter study confirmed these patterns: while ICCs were uniformly high, 95% repeatability limits again approached −1.1 to 1.4 D for Kmax (worse in more severe disease) and were very large for corneal astigmatism axis (worsening markedly in advanced KC) (117). This has direct implications for “progression criteria”: changes near common cutoffs (e.g., −1 D) can fall within measurement noise in many KC eyes. Building on repeatability data, the multicenter study proposed more conservative annual thresholds (e.g., larger Kmax and pachymetry changes) and emphasized combining multiple concordant indicators to reduce false-positive progression labeling (117). Clinically, axis-dependent planning should not rely on a single scan in moderate-to-advanced KC. As a pragmatic rule, at least three high-quality scans should be reviewed, and if the astigmatic axis differs by more than 5–10° across repeated scans, treatment planning should be repeated on another visit or cross-checked against manifest refraction, cone location, and topographic pattern before final surgical planning.

Severity matters: repeatability deteriorates as KC becomes more irregular. A dedicated analysis across KC grades showed that repeatability is relatively similar to controls in subclinical disease, but degrades in mild KC and worsens further in moderate KC, with particularly notable deterioration for Kmax and anterior astigmatism (repeatability limits increasing into ranges that can confound clinical decisions) (119). This severity dependence helps explain inconsistent axis readings and reinforces a pragmatic approach: in moderate-to-advanced KC, repeat measurements and trend confirmation are essential before acting on “small” changes. Building on this, our recent study used the MS-39 (high-resolution anterior segment OCT), an anterior segment OCT- and Placido-based tomographer, to establish updated, severity-stratified repeatability-based progression thresholds for KC (120).

Scan protocol and device interchangeability: do not mix-and-match casually. Even within the same platform, scan protocols can influence repeatability. A study comparing two Pentacam HR scan modes (25-3D vs. 50-Cornea Fine) found generally excellent ICCs but highlighted persistently poor repeatability for astigmatism axis and certain indices; Kmax repeatability limits remained around −1.0 D in both modes, while axis repeatability limits were extremely large, underscoring the need for cautious interpretation of axis outputs in KC (121). Across instruments, interchangeability is even more problematic: an evaluation of a new integrated aberrometry/keratometry system (iDesign 2) showed strong intrasession repeatability for keratometry in mild–moderate KC, yet limits of agreement vs. other devices were wide, and agreement worsened with steeper or more astigmatic corneas—arguing against substituting devices during follow-up or surgical planning (122).

Epithelial masking: KC epithelium can remodel to partially compensate stromal irregularity (123). A prospective study comparing measurements before and after epithelial removal demonstrated that keratometric/astigmatic metrics can shift after de-epithelialization, suggesting that preoperative “epithelium-on” maps may not fully represent the underlying stromal shape used to guide certain customized procedures (124). While this does not invalidate epithelium-on measurements for functional vision, it highlights a key caveat for cornea-regularizing ablation planning and for interpreting topographic changes around epithelium-off procedures.

Recent progress: OCT-based total corneal astigmatism and quantitative irregularity metrics (125). Advances in anterior segment OCT offer two complementary improvements. First, OCT-based net corneal astigmatism estimation (reconstructing anterior and posterior surfaces and deriving astigmatism from fitted models) can improve agreement with manifest cylinder in KC (125, 126). In a direct comparison, OCT-derived net astigmatism showed better accuracy than Pentacam when referenced to manifest refraction, and also demonstrated better repeatability under some settings; importantly, this work revealed a clinically relevant trade-off: pupil-centered smaller zones tended to better match manifest refraction (accuracy), whereas vertex-centered larger zones improved repeatability, emphasizing that “best” settings depend on the intended use (126). Second, OCT-enabled Fourier decomposition of corneal dioptric power can explicitly quantify components of irregular astigmatism (asymmetry and higher-order irregularity) and appears sensitive to early disease signatures—particularly on the posterior surface—thereby providing a structured framework to describe “irregular astigmatism” rather than treating it as an unmeasurable residual (127, 128). Fourier-derived irregularity components and Zernike-derived aberrations such as coma and trefoil are complementary but mathematically distinct descriptors, and should not be used interchangeably. In addition, for astigmatism-correcting procedures in KC, vector analysis (e.g., the Alpins method) may help guide surgical planning by accounting for both target-induced and surgically induced astigmatic change, although interpretation remains limited by irregular optics and poor axis repeatability.

Taken together, the evidence supports a “standardize and verify” approach. When clinical decisions depend on toric correction or total optical assessment, total corneal metrics, such as ray-traced or net corneal astigmatism, should be prioritized, and the selected algorithm should be clearly reported. Centration, whether pupil-, apex-, or vertex-centered, and analysis zone diameter should also be standardized and kept consistent across visits, because these settings can shift astigmatism vectors by clinically meaningful amounts in KC. For progression monitoring or axis-dependent surgical planning, decisions should rely on repeatability limits rather than intraclass correlation coefficients, and should be based on multiple high-quality scans with confirmation of trends rather than isolated borderline changes. Finally, because axis measurements are intrinsically less repeatable in KC, particularly in more advanced disease, the astigmatic axis should be interpreted as a consensus estimate derived from repeated measurements and cross-validation with corneal maps and irregularity patterns, rather than as a single definitive value.

## 4 Therapeutic refractive surgery for KC astigmatism: advances and outcomes

Building on the measurement framework above, contemporary surgical management of keratoconic astigmatism should not be viewed as a fixed sequence of procedures, but rather as an individualized strategy guided primarily by the patient's baseline CDVA, corneal thickness and stromal reserve, and the extent to which visual impairment is driven by irregular astigmatism and coma-dominant HOAs (3). In this context, CDVA provides a practical indicator of functional disease severity, corneal thickness determines the feasibility and safety of corneal tissue-modifying procedures, and the HOA profile helps distinguish eyes that may benefit more from corneal regularization than from purely refractive correction. Based on these principles, surgical management is best understood as a modular, phenotype-driven framework rather than a mandatory three-stage sequence. Stabilization of the ectatic process, most commonly with CXL, is indicated when progression is present; corneal regularization with customized surface ablation or ICRS/CAIRS may be selected when irregular astigmatism and coma-dominant HOAs are the principal causes of visual impairment; and correction of residual, relatively regular low-order astigmatism may be considered once corneal stability and sufficient optical regularity have been achieved, typically with toric phakic intraocular lenses or, when indicated, lens-based surgery (9). These components may be used individually or in combination, and not every patient requires all three. Biomechanically, these procedures act through different mechanisms and should therefore not be considered interchangeable. CXL primarily increases stromal stiffness to reduce the risk of further ectatic deformation, but its refractive effect is indirect and variable (7, 8). Customized surface ablation reshapes the anterior corneal surface to reduce asymmetric optical error, but removes stromal tissue and therefore requires conservative tissue limits and biomechanical stabilization (8). ICRS and CAIRS are additive intrastromal procedures that regularize the cornea by redistributing stromal stress and altering corneal arc length without tissue removal (10, 11), whereas toric phakic IOLs and lens-based procedures correct residual regular refractive error without removing corneal tissue or intentionally reshaping the cornea (13, 129).

### 4.1 Customized surface ablation

#### 4.1.1 Role in KC astigmatism

After ectatic progression has been controlled—most commonly with CXL—customized surface ablation provides a corneal regularization strategy aimed at reducing irregular astigmatism and coma-dominant HOAs, improving functional vision rather than pursuing emmetropia. In KC, the experienced “astigmatism” often reflects spatially heterogeneous optics and coma-like aberrations that are incompletely addressed by spectacle cylinder alone (3). Accordingly, modern protocols emphasize tissue-sparing regularization, typically by: (i) targeting irregularity/HOAs (often coma) with limited or no refractive correction, (ii) imposing strict ablation caps (commonly ≤50 μm), and (iii) combining ablation with CXL either simultaneously or sequentially to reduce the risk of biomechanical destabilization (8, 15, 16).

Current approaches include: (1) topography-guided PRK (TG-PRK; including T-CAT), which has the strongest evidence base; (2) wavefront-guided PRK (corneal vs. ocular wavefront), often implemented as HOA-only tissue-saving algorithms; and (3) “regularization” platforms such as central corneal regularization (CCR) that use small optical zones with large transition zones to minimize stromal removal while improving central optics (15, 17–19).

#### 4.1.2 Indications and limitations

Customized ablation is best suited for patients whose primary limitation is HOA-driven image degradation (ghosting, glare, fluctuating vision) despite appropriate refraction and/or contact lens challenges, and in whom corneal clarity is adequate (no visually significant central scarring) (1, 8). In practical terms, this approach is generally most appropriate for eyes with mild to moderate functional impairment, typically with a CDVA of approximately 0.6 to 0.9 (decimal), in which useful visual potential remains but optical quality is significantly compromised by irregular astigmatism and coma-dominant HOAs. In most studies, candidates have documented progression by tomographic/keratometric and/or refractive criteria and are therefore treated within a CXL-plus framework (8).

Across platforms, the main gatekeeper is corneal thickness and residual stromal reserve. Common thresholds include: (i) thinnest corneal thickness (TCT) ≥ 450 μm to allow limited ablation while maintaining the ≥400 μm requirement for safe epithelium-off CXL, and (ii) a maximum ablation depth cap (typically ≤50 μm) for KC regularization (15, 17, 20–22). These cut-offs should be interpreted as pragmatic safety conventions rather than absolute biomechanical guarantees, particularly in younger patients or thinner corneas, where the long-term effects of tissue removal remain incompletely defined. Accordingly, this strategy should be considered only in eyes with sufficient corneal thickness to safely tolerate tissue removal, and several studies implement this pragmatically by allocating eyes to CXL alone if predicted post-ablation thickness would fall below safety limits (17, 21).

Technique selection should be individualized to the corneal phenotype and data quality. In general, TG-PRK is preferred when corneal irregularity dominates and high-quality corneal topography is available; most protocols prioritize optical regularization rather than full refractive correction by setting spherical correction to zero or partial treatment, limiting cylinder correction, and capping ablation depth (15, 17, 20, 21). Ocular wavefront-guided (OWG) or HOA-only approaches may be advantageous when whole-eye wavefront measurements are repeatable; one pragmatic strategy is to select the plan with the lowest predicted maximum stromal removal when comparing OWG with corneal wavefront/topography-based options (16, 18). CCR may be considered when the main goal is central optical regularization with minimal tissue consumption, typically using a very small optical zone centered on the cone/apex with a large transition zone (19).

Regarding timing, the overall efficacy of customized PRK + CXL is supported by meta-analytic evidence (8), whereas the specific haze comparison between same-day and delayed/sequential PRK + CXL is based mainly on large retrospective series (15). Same-day PRK followed by CXL has been reported to achieve favorable outcomes with generally acceptable haze profiles (15); however, superiority in reducing haze remains insufficiently validated (129), and PRK + CXL may produce more pronounced or prolonged haze than CXL alone (23).

#### 4.1.3 Results

Across the included literature, the clinical value of PRK + CXL is supported primarily by improvements in visual acuity and refractive outcomes, whereas topographic and aberrometric changes should be interpreted as evidence of optical regularization rather than stand-alone proof of functional visual improvement (21, 24–26). The 2025 meta-analysis of 8 nonrandomized studies including 731 eyes showed that customized PRK + CXL achieved better outcomes than CXL alone, with significant improvements in UDVA and CDVA, reductions in refractive cylinder and mean keratometry, and reductions in coma and total HOAs (8).

At the individual-study level, manifest cylinder generally improved but remained protocol-dependent. Zhang and Chen reported a reduction from −3.75 ± 1.53 D to −3.07 ± 1.96 D at 12 months (25), whereas Iqbal et al. found that the astigmatic component decreased from 1.83 ± 0.69 D to 1.15 ± 0.26 D, with a mean astigmatic correction of 0.86 ± 0.34 D (27). In contrast, the Bharat Protocol did not significantly improve topographic cylinder, despite regularization of Kflat/Ksteep/Kmax from 46.94/49.75/55.99 D to 43.89/47.21/51.76 D (26).

These findings suggest that PRK + CXL is more consistently associated with corneal normalization than with precise astigmatic neutralization. In the 2021 study, K_apex decreased from 57.23 ± 5.09 D to 53.13 ± 4.47 D, while 6 mm HOA RMS and coma RMS decreased from 2.25 ± 0.84 to 1.57 ± 0.85 μm and from 1.82 ± 0.78 to 1.14 ± 0.79 μm, respectively (25). Dai et al. also reported greater Kmax reduction with TG-PRK + CXL than with CXL alone, accompanied by reductions in 6 mm corneal HOAs and vertical coma (21).

Refractive predictability remains moderate and highly protocol-dependent. In one WG-PRK series, 92.6% of eyes were within ± 1.0 D manifest refraction spherical equivalent (MRSE) and 81.5% had residual refractive cylinder within ± 1.0 D at 12 months (28), whereas other regularization-oriented protocols reported 76.5% within ± 1.0 D spherical equivalent (SE) (24), 22% within ± 0.50 D SE, and 38% achieving <0.5 D astigmatic deviation from target (29). In Dai et al., long-term Alpins analysis showed mean target-induced astigmatism (TIA) 0.74 × 173°, surgically induced astigmatism (SIA) 0.18 × 105°, and difference vector (DV) 0.87 × 177°, with no significant TIA–SIA correlation (21). Clinically, this indicates limited and unpredictable vectorial astigmatic neutralization, so Alpins indices should be interpreted as measures of treatment accuracy rather than evidence of refractive precision comparable to conventional astigmatic laser correction. Accordingly, PRK + CXL should be viewed mainly as a corneal normalization strategy that may secondarily reduce manifest cylinder, rather than as a highly precise astigmatic refractive correction procedure.

### 4.2 Intracorneal ring segments

#### 4.2.1 Role in KC astigmatism

ICRS are synthetic, arc-shaped implants placed in the deep corneal stroma to redistribute biomechanical stress and improve keratoconic vision primarily through corneal regularization, flattening of the ectatic cone, and reduction of irregular astigmatism and corneal asymmetry (30–33). In current practice, ICRS should be viewed primarily as a corneal regularization procedure—aimed at reducing irregularity and improving optical quality—rather than a purely refractive, cylinder-neutralizing intervention. Importantly, despite the long clinical experience with ICRS and the progressive refinement of nomograms, their refractive predictability remains limited, and outcomes still depend substantially on baseline corneal phenotype and disease severity (34).

#### 4.2.2 Indications and limitations

Patient selection is central to obtaining meaningful astigmatism-related benefit from ICRS. Rather than being based on CDVA alone, ICRS candidacy should integrate cone morphology, cone location, asymmetry pattern, corneal clarity, stromal thickness at the intended tunnel site, and contact-lens intolerance or poor lens tolerance. Reduced spectacle CDVA remains clinically relevant for risk–benefit assessment; based on previous outcome analyses, eyes with spectacle CDVA worse than 0.6 decimal may have greater potential for visual gain, whereas eyes with good preoperative corrected vision are less favorable candidates because of the higher risk of postoperative CDVA loss (10, 34). In this context, ICRS may be particularly valuable in eyes in which customized surface ablation is not feasible because of insufficient corneal thickness or inadequate stromal reserve, since they can improve corneal regularity without requiring tissue removal. Surgical planning typically selects the number, thickness, and arc length of segments according to manufacturer nomograms (Table 2), with the corneal incision placed at the steepest meridian based on corneal topography (10). Practical exclusion criteria include insufficient stromal thickness at the intended tunnel site, relative to manufacturer safety thresholds, usually <400 μm, severe central scarring, or a history suggestive of prior corneal hydrops (10). Although ICRS implantation is generally considered safe, particularly with femtosecond-laser tunnel creation, complications may include segment decentration or migration, extrusion, infectious keratitis, corneal melting, and explantation; perforation is uncommon with femtosecond techniques (<0.6%), infectious keratitis has been reported in <0.1%, and explantation rates vary widely from approximately 1% to 30% (35–42). Even after successful implantation, residual irregular astigmatism and contact-lens dependence may persist, and worsening refractive cylinder during follow-up may serve as a warning sign for late extrusion (43).

**Table 2:** Technical specifications of commonly used intracorneal ring segments (ICRS).

Model	Arc length (°)	Thickness (µm)	Cross-section	Optical zone (mm)	Inner diameter (mm)	Outer diameter (mm)
Intacs	150, 210	210, 230, 250–450 (50 µm steps)	Hexagonal	7	6.77	8.1
Intacs SK	90, 130, 150	210, 250–500 (50 µm steps)	Elliptical	6	6	7
Keraring	90, 120, 150, 160, 210, 340, 355	150–350 (50 µm steps)	Triangular	5.0, 5.5, 6.0	5	6
Ferrara	90, 100, 110, 120, 130, 140, 150, 160, 170, 180, 190, 210, 320	150–350 (50 µm steps)	Triangular	5.0, 6.0	4.4	5.6
Myoring	360	200–320	Triangular	No data	5.00–8.00	6.00–9.00

#### 4.2.3 Results

In the most recent systematic review and meta-analysis of asymmetric ICRS, which included 7 observational studies and 435 eyes, implantation resulted in significant but heterogeneous improvements in topographic astigmatism (MD 2.04 D; 95% CI, 1.51–2.58; *I*^2^ = 66%), refractive astigmatism (MD −2.52 D; 95% CI, −3.08 to −1.95; *I*^2^ = 61%), Kmax (MD 4.42 D; 95% CI, 3.24–5.60; *I*^2^ = 50%), Kmean (MD 2.92 D; 95% CI, 1.94–3.91; *I*^2^ = 70%), and comatic aberration (MD 0.56 μm; 95% CI, 0.29–0.82; *I*^2^ = 42%) (30). Because these estimates are derived from observational pre–post studies with moderate-to-high heterogeneity, they should be interpreted cautiously and not as definitive comparative treatment effects (30). At the individual-study level, de Sanctis et al. reported that Keraring implantation guided by a topographic pattern-based nomogram reduced mean refractive cylinder from 4.99 ± 1.89 D to −2.31 ± 1.47 D, while mean and maximum keratometry decreased by 2.10 ± 1.42 D and 3.02 ± 3.68 D, respectively; notably, corneal HOA RMS decreased from 3.296 ± 1.180 μm to 2.192 ± 0.919 μm and vertical coma from −2.656 ± 1.189 μm to −1.427 ± 1.024 μm, indicating that astigmatic improvement was accompanied by substantial optical regularization (31). Likewise, in duck-type KC treated with AJL PRO + asymmetric ICRS, refractive cylinder improved from −3.73 ± 2.16 D to −1.99 ± 1.10 D and spherical equivalent from −5.51 ± 3.68 D to −1.42 ± 2.55 D, together with a reduction in total corneal HOA RMS from 1.93 ± 0.89 μm to 1.69 ± 0.78 μm and coma from 1.62 ± 0.81 μm to 0.99 ± 0.59 μm, again suggesting that the main functional benefit derives from simultaneous reduction of both regular and irregular optical error (32). With regard to predictability, ICRS is more suitable than PRK + CXL for vector analysis, yet its refractive accuracy remains moderate and strongly dependent on cone morphology, nomogram selection, and segment alignment (31, 32, 44). In the Alpins vector study by Abozaid and Abdalla, combined Keraring implantation and CXL yielded a manifest astigmatic difference vector of 2.10 D and a correction index of 1.03, but only 50% of eyes had an angle of error within ± 5°, while corneal astigmatism showed poorer predictability with an *R*^2^ of only 0.07 for the SIA–TIA relationship (44). Similarly, in the AJL PRO + series, 63.6% of eyes had an angle of error within ± 15°, and 55% achieved a postoperative MRSE within ± 1.50 D (32). Long-term data further indicate that the most durable effect of ICRS is topographic and aberrometric stabilization rather than perfect refractive precision: in the 5-year INTACS study, anterior SimK flattened from 50.667 ± 2.581 D to 47.733 ± 2.434 D and coma RMS decreased from 2.484 ± 1.548 to 1.642 ± 1.163, whereas cylinder remained largely unchanged over time, supporting the view that ICRS should be regarded primarily as a corneal remodeling procedure with secondary astigmatic benefit rather than as a highly precise cylinder-correcting intervention (45).

### 4.3 Corneal allogeneic intrastromal ring segments

#### 4.3.1 Role in KC astigmatism

**Figure 2:**
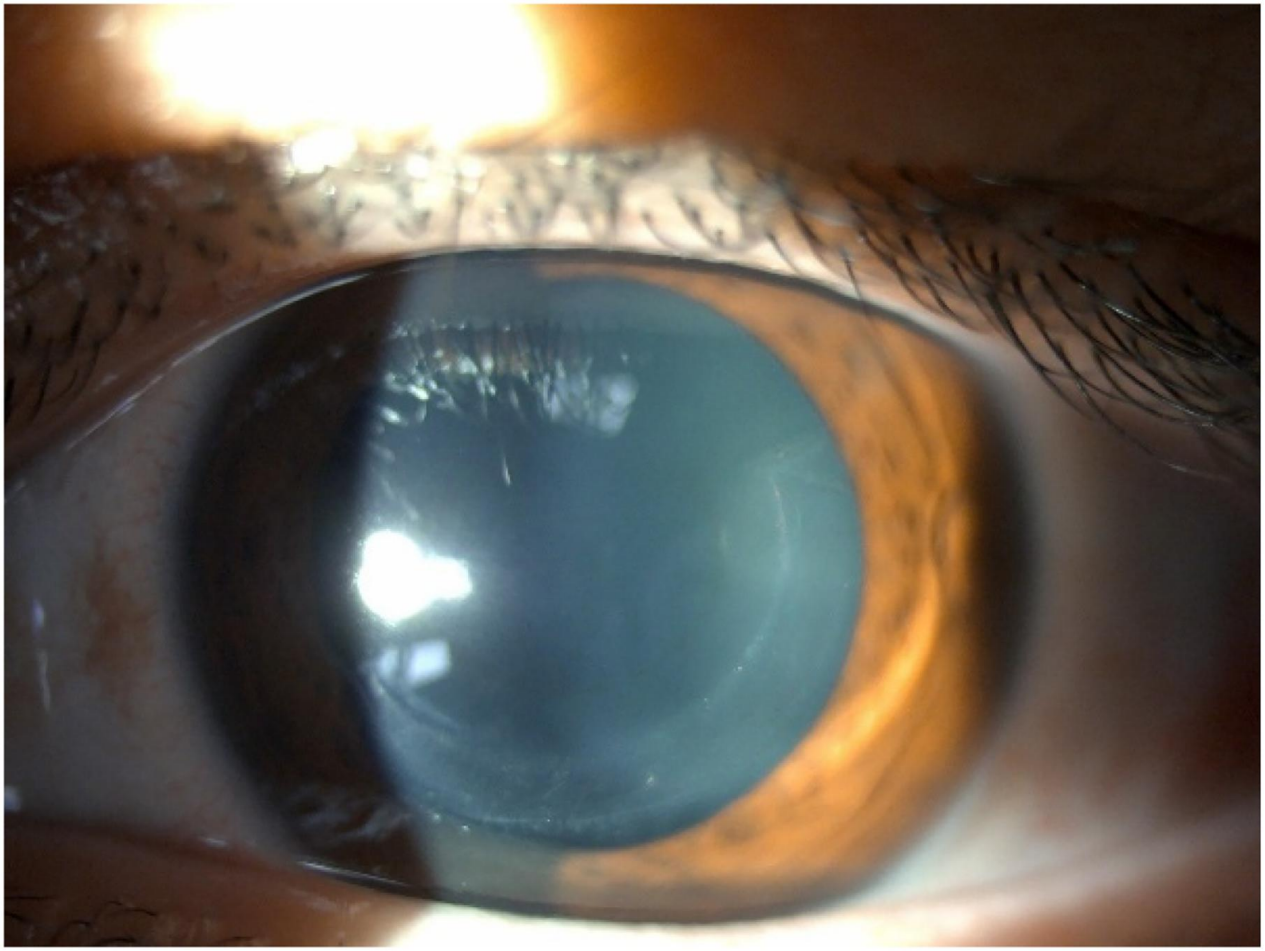
Corneal allogeneic intrastromal ring segments.

ICRS remain the more established corneal ring technique, with longer clinical experience and more standardized nomogram-based planning, whereas CAIRS have emerged in recent years as a tissue-based alternative for corneal regularization in KC (Figure 2 and Table 3) (46–67). Similar to synthetic ICRS, CAIRS should be regarded primarily as a corneal regularization procedure rather than a technique for direct refractive cylinder cancellation (46, 61, 68). Compared with synthetic segments, CAIRS have several biologically plausible advantages, including improved stromal biocompatibility, absence of permanent synthetic material, a refractive index closer to native corneal tissue, and potential stromal volume augmentation. However, these advantages remain theoretical or early clinical observations rather than validated evidence of superior long-term visual or refractive outcomes. In addition, unlike synthetic segments with fixed geometries, CAIRS can be customized in arc length, width, and thickness, but no universally accepted nomogram currently exists for selecting graft geometry or combining CAIRS with CXL (49, 58, 66, 68, 69).

**Table 3:** Summary of recent evidence on CAIRS for the management of keratoconus.

Author and Year	Study design	Case number	Follow-up	CAIRS calculation	CAIRS preparation	CXL (yes/no)	Diagnosis
Jacob et al. 2018 (46)	Prospective interventional case series	24 eyes (20 patients)	Mean 11.6 ± 3.6 months (range 6–18)	Uniform size; same diameter/arc length for all	Trephine (double-bladed Jacob CAIRS trephine); donor corneal rims	Yes – intraoperative A-CXL or A-CACXL	Keratoconus
Haciagaoglu et al. 2023 (47)	Retrospective case series	44 eyes (32 patients)	6 months	Istanbul Nomogram (inner Ø 4 mm; outer Ø 7.5 mm; depth 200 µm; cone-guided)	Femtosecond laser tunnel (iFS 150 kHz); KeraNatural™ sterile allograft	No (deferred if progression)	Keratoconus
Nacaroglu et al. 2023 (48)	Retrospective case series	65 eyes (49 patients)	12 months	Istanbul Nomogram (inner Ø 4 mm; outer Ø 7.5 mm; −35% depth; single 160° over cone)	Femtosecond laser tunnel (iFS 150 kHz); KeraNatural^®^	Preoperative CXL only (mean 60.9 ± 42.1 months before)	Keratoconus
Jacob et al. 2023 (49)	Prospective interventional case series	32 eyes (29 patients)	≥12 months (mean ∼1 year)	Individual topography-guided; tapered/variable volume per Barraquer's law	Trephine + CAIRS Customizer™; donor corneoscleral rims; femto tunnel at ∼50% depth	Yes – intraoperative A-CXL/A-CACXL (31/32 eyes)	Keratoconus with decentered asymmetric cone
Kirgiz et al. 2024 (50)	Retrospective case series	23 eyes (23 patients)	6 months	Modified Istanbul (inner Ø 4.5 mm; outer Ø 7.75 mm; depth 200 µm)	Sequential trephination (8 mm → 6 mm punch); femto tunnels (VisuMax)	Partly – prior CXL in 65% (≥12 months); no simultaneous CXL	Keratoconus
Mazzotta et al. 2024 (51)	Case report/technical note (*n* = 2)	2 eyes (2 patients)	Up to 3 months	Modified MS39–Alfonso integrated; MZ personalized nomogram	All-femtosecond laser–cut trapezoid segments; 1.5 mm wide; 300–460 µm thick	No corneal CXL; A-CXL applied to segments pre-implant	Keratoconus
Yucekul et al. 2024 (52)	Retrospective comparative case series	67 eyes (47 patients)	6 months	Istanbul Nomogram (inner Ø 4 mm; outer Ø 7.5 mm; depth 200 µm; single 160° segment)	Femtosecond laser (iFS 150 kHz); KeraNatural allograft; annular tunnel concentric to cone	30 eyes with prior CXL; 37 without prior CXL	Keratoconus
Bteich et al. 2025 (53)	Observational case series (design not explicitly stated)	20 eyes (15 patients)	12 months	Surgeon-specific (AUBMC); optical zone 6.0 mm; depth 250–300 µm; 20% double segments	Femtosecond laser–cut (LDV Z8); donor rim in artificial AC; 500/750 µm thickness options	No (neither simultaneous nor staged)	Corneal ectasia
Keskin Perk et al. 2025 (54)	Retrospective case series	62 eyes (49 patients)	Mean 23.2 ± 13.5 months (6–36); visits at 1, 6, 12, 36 months	Istanbul Nomogram (inner Ø 4 mm; outer Ø 7.5 mm; depth 200–250 µm; ∼160°; 1 mm width)	KeraNatural sterile allograft; trimmed to ∼160°; femto tunnels (iFS)	No (prior CXL and double-ring cases excluded)	Keratoconus (treatment)
Benz et al. 2025 (55)	Retrospective interventional case series (single-surgeon)	17 eyes (16 patients)	Mean 141.1 ± 79.9 days (range 30–338)	Modified Istanbul; inner Ø 4–5 mm; outer Ø 7.2–8.2 mm; tunnel width 1.6 mm; depth 200–250 µm (pachymetry-guided)	Femtosecond laser (Ziemer Z6); KeraNatural sterile arc implant; OCT-guided centration; no sutures	1 eye had prior CXL; 9 eyes underwent adjunctive CXL ≥ 1 month post-CAIRS	Keratoconus
Bteich et al. 2023 (66)	Interventional case series (4 eyes of 2 patients)	4 eyes (2 patients)	6 months	Asymmetric femtosecond laser–cut customized segments (size/shape/arc/thickness variable)	Femtosecond laser (LDV Z8) cutting on donor cornea mounted on artificial anterior chamber; proprietary software; dehydration on circular mold	No simultaneous CXL reported	Keratoconus with non-coinciding astigmatism and coma axes
Bteich et al. 2023 (56)	Retrospective study	52 eyes (39 patients)	Mean 6.9 ± 5.2 months (median 5; range 3–24)	Modified PMMA-ICRS nomogram: incision on steep meridian; 1 segment for decentered cones/2 for centered; segment size 500 or 750 µm & depth 250 or 300 µm per Kmax–Kcenter/SimK/MRSE; arc length 160–170°; optical zone 5–6 mm.	Donor corneoscleral rims; epi/endo scraped; trephined (Jacob CAIRS) to 500/750 µm, bisected; mounted on 5/6 mm PVA disc; LDV Z8 femto tunnels (inner Ø 5/6 mm; outer Ø 6.8/7.8 mm; width 900 µm; depth 250–300 µm); Bowman's layer toward apex; no sutures; bandage lens.	No — stand-alone CAIRS; CXL deferred ≥ 3 months; any post-CXL data excluded.	Keratoconus (47 eyes) or corneal ectasia after laser vision correction (5 eyes)
Coscarelli et al. 2024 (57)	Single-arm cohort prospective study	3 eyes (3 patients)	Mean 6.01 ± 1.02 months	Donut-shaped 360° CAIRS; femtosecond channel inner Ø 5.40 mm, outer Ø 8.00 mm; 30° angulation; 50% of thinnest pachymetry at desired location (optical zone 5.4–8.0 mm).	Donor stromal tissue from DALK remnants; double-bladed trephine 7.50 & 9.75 mm; epithelium/Descemet/Dua/endothelium removed; LDV (Ziemer) femtosecond channel; implanted 360° with McPherson forceps & Sinskey hooks; tips left near entry incision.	No — stand-alone donut-shaped CAIRS (no simultaneous CXL reported).	Keratoconus
Mechleb et al. 2024 (58)	Case series	10 eyes (10 patients)	1 month	Preop plan: depth 250 µm (regardless of thickness); 5 mm optical zone; arc length per refraction (up to 180°) with incision on steep meridian guided by refraction/coma; CAIRS thickness 400 µm for all; width per Bteich nomogram (wider in very severe KC, e.g., 1,200 µm).	Donor corneas (organ culture); FS200 femtosecond multiple concentric lamellar cuts at 400 µm using a pressurized artificial anterior chamber (200 mmHg); cuts marked for centration; annuli air-dried; tunnels created at 5 mm OZ, 250 µm depth, sized 100 µm larger than CAIRS; dehydrated segments manually inserted and positioned (Sinskey); standard postop drops.	No simultaneous CXL stated; prior CXL NR.	Keratoconus
Hayashi et al. 2025 (59)	Prospective case series	5 eyes (5 patients)	Mean 6.4 ± 2.9 months	Istanbul nomogram; cone axis by CAIRSplan; tunnel inner Ø 5.0 mm; width ∼3 mm; implantation depth ∼200–215 μm; graft length to cover cone	Manual pocket (guarded knife + Hayashi CAIRS spatula); donor punched with double-bladed Jacob CAIRS trephine; AC air pressurization; Flieringa ring; iOCT; 10–0 nylon; standard postop meds	No simultaneous CXL reported;	Keratoconus (Amsler–Krumeich stage 2–4; decentered cones)
Mechleb et al. 2025 (60)	Retrospective observational case series	79 eyes (71 patients)	6 months	Depth 250 µm for all; 5 mm optical zone; arc length 90–160° per refraction/severity; incision on steep meridian (centered between coma axis and cone apex if axes differ); width by Bteich nomogram (up to 1,200 µm for Kmax > 75 D)	Femtosecond laser preparation on donor grafts (anterior lamellar mode); multiple concentric cuts at 400 µm to create annuli; air-dried; intrastromal tunnels per plan; dehydrated CAIRS manually inserted; standard postop antibiotics/steroids	NR (simultaneous/prior CXL not specified)	Keratoconus; post–corneal refractive surgery ectasia
Asfar et al. 2024 (61)	Retrospective cohort with propensity score matching (single surgeon, single center)	34 CAIRS eyes (31 patients), matched 1:1 to 34 ICRS eyes	Mean 9.2 ± 4.1 months (CAIRS); visits at 1 week, 1, 3, 6, 12 months; last FU defined before any CXL	Nomogram adapted from PMMA ICRS; 1 segment (160° arc) at 6 mm OZ; segment size 500 or 750 µm based on Kmax–Kcenter/SimK/MRSE; tunnel depth 250–300 µm; incision on steep meridian	Donor corneoscleral rims (epi/endo removed); double-bladed Jacob's trephine (500/750 µm) → annuli (ID/OD 6.50/8.00 or 7.75/8.75 mm) → bisected/trimmed to 160°; LDV Z8 tunnels (inner Ø 5.8 mm; outer Ø 7.6 mm; width 900 µm; depth 250–300 µm)	No concomitant CXL; prior CXL excluded; if CXL performed later, patient exited and last pre-CXL visit used	Keratoconus
Jacob et al. 2025 (67)	Retrospective interventional case series (post-implantation adjustment)	13 eyes (13 patients)	Three time points: pre-implantation, post-implantation, post-adjustment (specific months NR)	Adjustment planned from AS-OCT: modify arc length/width/thickness, reposition along channel, or optical-zone correction (e.g., reduce OZ)	Jacob CAIRS instruments; curved Y-rod/manual tunnelizer to open/free segment; reverse Sinskey for explant/re-implant; double-bladed Jacob trephine if extra segment required	10 eyes had simultaneous CXL at initial surgery; 3 eyes had prior CXL (no CXL at adjustment)	11 keratoconus, 1 post-LASIK ectasia, 1 pellucid marginal degeneration
Awwad et al. 2025 (62)	Retrospective pilot study (validation of technique)	33 eyes (29 patients)	Up to 3 months (OCT at 1 wk, 1 mo, 3 mo)	Donor corneoscleral rims; epithelium/endothelium removed; trephined into annular rings; halved; placed in titanium mold on polyethylene drape over USB-powered cup heater (55 °C) for 5 min	Nomogram as previously described by the authors; single segment (750 µm Jacob trephine); femtosecond tunnels 5.8–7.6 mm, width 900 µm, depth 250–300 µm	No simultaneous CXL; CXL deferred ≥ 3 months; post-CXL data excluded	Keratoconus
Yakut et al. 2025 (64)	Prospective observational single-center study	35 eyes (27 patients)	12 months	Istanbul nomogram; single 160° segment; tunnel inner Ø 4.0 mm, outer Ø 7.5 mm; depth 250 µm	KeraNatural™ (ready-to-use sterile allograft CAIRS); femtosecond tunnel with iFS 150 kHz (Intralase)	NR (simultaneous/prior CXL not specified)	Keratoconus (advanced)
Colak et al. 2026 (65)	Retrospective case series	6 eyes (5 patients)	6 months–3 years (range)	Istanbul nomogram; dual opposing segments (for relatively symmetrical cones); tunnel inner Ø 4.0 mm, outer Ø 7.5 mm; depth 200–250 µm	KeraNatural (Lions VisionGift); femtosecond tunnel iFS 150 kHz; segments manually shaped; soaked in BSS before implantation	Mixed — 1 patient had prior CXL (2 years pre-op); no intraoperative CXL	Keratoconus (relatively symmetrical cones)

AC, anterior chamber; A-CACXL, accelerated contact lens–assisted CXL; A-CXL, accelerated CXL; AS-OCT, anterior segment optical coherence tomography; AUBMC, American University of Beirut Medical Center; BSS, balanced salt solution; CAIRS, corneal allogeneic intrastromal ring segments; CXL, corneal collagen cross-linking; DALK, deep anterior lamellar keratoplasty; endo, endothelium; epi, epithelium; FS, femtosecond laser; FU, follow-up; ID, inner diameter; ICRS, intracorneal ring segments; iOCT, intraoperative optical coherence tomography; KC, keratoconus; Kcenter, keratometry at the corneal center; Kmax, maximum keratometry; LASIK, laser-assisted *in situ* keratomileusis; mo, month; MRSE, manifest refraction spherical equivalent; MZ, Mazzotta–Zagari; NR, not reported; OCT, optical coherence tomography; OD, outer diameter; OZ, optical zone; PMMA, polymethyl methacrylate; postop, postoperative; preop, preoperative; PVA, polyvinyl alcohol; SimK, simulated keratometry; USB, universal serial bus; wk, week.

#### 4.3.2 Indications and limitations

From an astigmatism-management perspective, CAIRS candidacy should be grounded in the cornea's regularization potential. Similar to synthetic ICRS, CAIRS is generally considered reasonable in eyes without dense central corneal scarring, whereas eyes with substantial central scarring are more appropriately directed toward deep anterior lamellar keratoplasty (DALK) or penetrating keratoplasty (PKP) (1). Moreover, extremely ectatic corneas may lie beyond the effective range of CAIRS. For example, a small case series reported no visual acuity improvement in advanced-stage KC with anterior topographic Kmax > 75 D, suggesting that CAIRS may not reliably substitute keratoplasty in severely ectatic eyes (58).

A key unresolved variable is the role and timing of CXL. One retrospective comparative study reported larger improvements in eyes without prior CXL than in previously cross-linked eyes (52). Although the groups were comparable in the reported preoperative visual, refractive, keratometric, and pachymetric parameters, the retrospective nonrandomized design does not exclude residual confounding or unmeasured differences in patient selection. Therefore, the possibility that prior CXL attenuates the regularizing effect of CAIRS should be considered hypothesis-generating rather than established. Whether CAIRS can influence disease progression—particularly in younger patients—and whether preoperative or simultaneous CXL is beneficial or mandatory remain unanswered questions requiring longer-term and comparative studies.

Current CAIRS planning remains heterogeneous. Published techniques vary in tunnel creation, including femtosecond-assisted vs. manual or trephine-based approaches; graft preparation, including fresh, dehydrated, or sterile allogeneic tissue; and segment geometry (Table 3). Together with the unresolved role of CXL noted above, these variations limit direct comparison across studies and prevent firm technique-specific or nomogram-based recommendations.

Available short-term evidence suggests a generally favorable CAIRS safety profile, with reported segment subluxation in 0.7%, extrusion in 0.5%, loss of one CDVA line in 0.5%, acute rejection requiring explantation in 0.2%, and mild implant opacification or deposits in 4.1% (47–50, 54, 56, 58). However, refractive unpredictability, persistent irregular astigmatism, graft-related reactions, and late explantation remain clinically relevant concerns, and longer follow-up is required to define the true late adverse-event profile (70, 71).

#### 4.3.3 Results

Current CAIRS evidence is encouraging but remains immature, as most available studies are retrospective, single-arm, single-center series with heterogeneous techniques and relatively short follow-up. Therefore, meta-analytic estimates should be interpreted cautiously. In the 2025 systematic review and meta-analysis including 14 clinical studies and 442 eyes, CAIRS significantly improved CDVA by 0.37 logMAR and UDVA by 0.43 logMAR, improved spherical equivalent by 4.59 D, reduced Kmax by 4.49 D, and decreased total HOAs by 0.33 μm, with only one severe adverse event (0.2%) reported as acute rejection requiring explantation (11). These findings were reinforced by a 2026 systematic review and meta-analysis of 18 studies including 567 eyes, which showed that at 1 year, UDVA improved by 0.39 logMAR, CDVA by 0.34 logMAR, Kmean decreased by 3.59 D, and Kmax by 3.73 D, with no clear superiority of any specific graft-preparation technique or adjunctive cross-linking strategy (72). At the individual-study level, the 2023 comprehensive segmental tomography study of 52 eyes demonstrated improvement in UDVA from 0.97 ± 0.47 to 0.56 ± 0.25 logMAR and CDVA from 0.56 ± 0.19 to 0.22 ± 0.21 logMAR, accompanied by a reduction in Kmax from 58.09 to 52.33 D and mean K from 48.92 to 44.32 D at a mean follow-up of 6.9 months (56). In the customized CAIRS series for decentered asymmetric cones, refractive cylinder improved from −3.97 ± 1.34 D to −2.78 ± 1.59 D, front-surface astigmatism from 5.23 ± 1.44 D to 4.30 ± 1.67 D, Kmax from 58.9 ± 5.54 D to 54.95 ± 4.71 D, HOA RMS from 2.85 ± 0.78 to 2.59 ± 0.80 μm, and vertical coma from −2.09 ± 0.96 to −1.40 ± 1.21 μm; moreover, 27 of 32 eyes gained at least 2 lines of UDVA, supporting the concept that CAIRS can simultaneously reduce both regular and irregular optical error (49). Similarly, in the 2023 1-year series using sterile allogeneic ring segments, spherical equivalent improved from −8.82 ± 4.57 D to −3.45 ± 4.81 D at 6 months, while Kmax decreased from 59.20 ± 7.66 D to 55.83 ± 6.96 D at 6 months and 55.63 ± 6.14 D at 12 months, with significant improvement also reported in corneal astigmatism and average keratometry (48). However, refractive predictability remains less rigorously characterized than with synthetic ICRS, because no universally adopted nomogram exists and vector-analysis metrics or percentages within ± 0.50 D or ± 1.00 D are still rarely reported (56, 72). Available comparative evidence nevertheless suggests that outcomes may vary with prior CXL status and segment design. Yucekul et al. reported greater improvements in CDVA, K1, and Kmean in eyes without prior CXL than in previously cross-linked eyes (52). Although the reported preoperative visual, refractive, keratometric, and pachymetric parameters were comparable between groups, the retrospective nonrandomized design does not establish that prior corneal stiffening directly attenuates the effect of CAIRS. Separately, the 2024 propensity score–matched comparison with synthetic ICRS found broadly comparable overall improvements but a higher proportion of CAIRS eyes gaining two or more Snellen lines of CDVA (60% vs. 31.58%, *P* = 0.04), together with a greater reduction in vertical coma (61). Given the small sample sizes and phenotypic complexity of KC, these findings remain hypothesis-generating and require confirmation in larger prospective comparative studies. Taken together, the current evidence supports CAIRS as a biologically integrated corneal remodeling strategy that is particularly effective in improving corneal regularity, keratometric steepness, coma-driven optical distortion, and secondarily refractive astigmatism, while its true refractive predictability remains promising but not yet fully standardized (11).

### 4.4 Toric phakic IOLs

#### 4.4.1 Role in KC astigmatism

After disease stabilization, most commonly with CXL, and when needed after corneal regularization with customized surface ablation or ICRS/CAIRS, toric phakic intraocular lenses (pIOLs) can serve as a refractive finishing step in KC (73, 129). Their principal role is to correct residual low-order refractive error, particularly myopia and relatively regular astigmatism, without removing corneal tissue or further altering corneal biomechanics (73, 129). In this respect, toric pIOLs differ fundamentally from corneal procedures: they do not regularize the ectatic cornea and do not directly address the irregular astigmatism or coma-dominant HOAs that often limit vision in more advanced disease (74). Accordingly, their best indication is not primary optical rehabilitation of an irregular cornea, but rather precise correction of the residual regular refractive component once sufficient corneal stability and regularity have been achieved.

**Figure 3:**
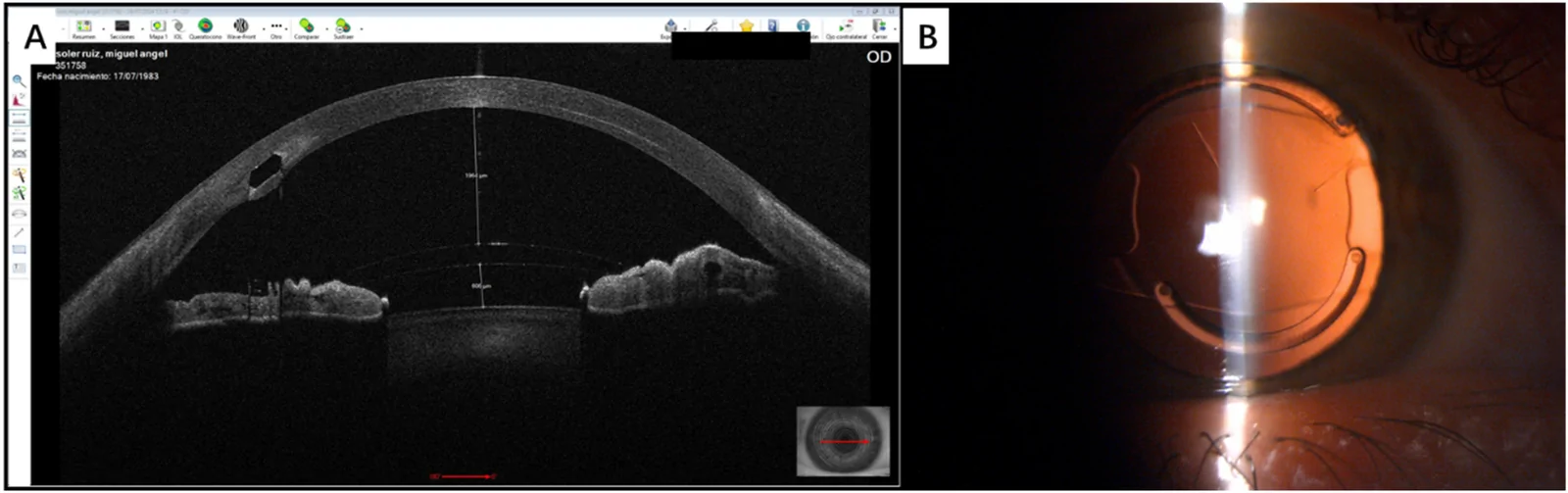
A 42-year-old male with keratoconus previously treated with intracorneal ring segment (ICRS) implantation and photorefractive keratectomy (PRK) for corneal remodeling, who subsequently underwent anterior chamber iris-fixated intraocular lens (artisan) implantation for residual ametropia. Postoperatively, uncorrected distance visual acuity (UDVA) reached 0.90 and improved to a corrected distance visual acuity (CDVA) of 1.0 with minimal residual astigmatism, while intraocular pressure remained within normal limits. **(A)** Anterior segment OCT showing the ICRS positioned within the corneal tunnel and a stable anterior chamber configuration with a well-fixated iris-claw intraocular lens. **(B)** Slit-lamp photograph confirming appropriate iris fixation of the intraocular lens and a quiet anterior segment without signs of inflammation.

**Figure 4:**
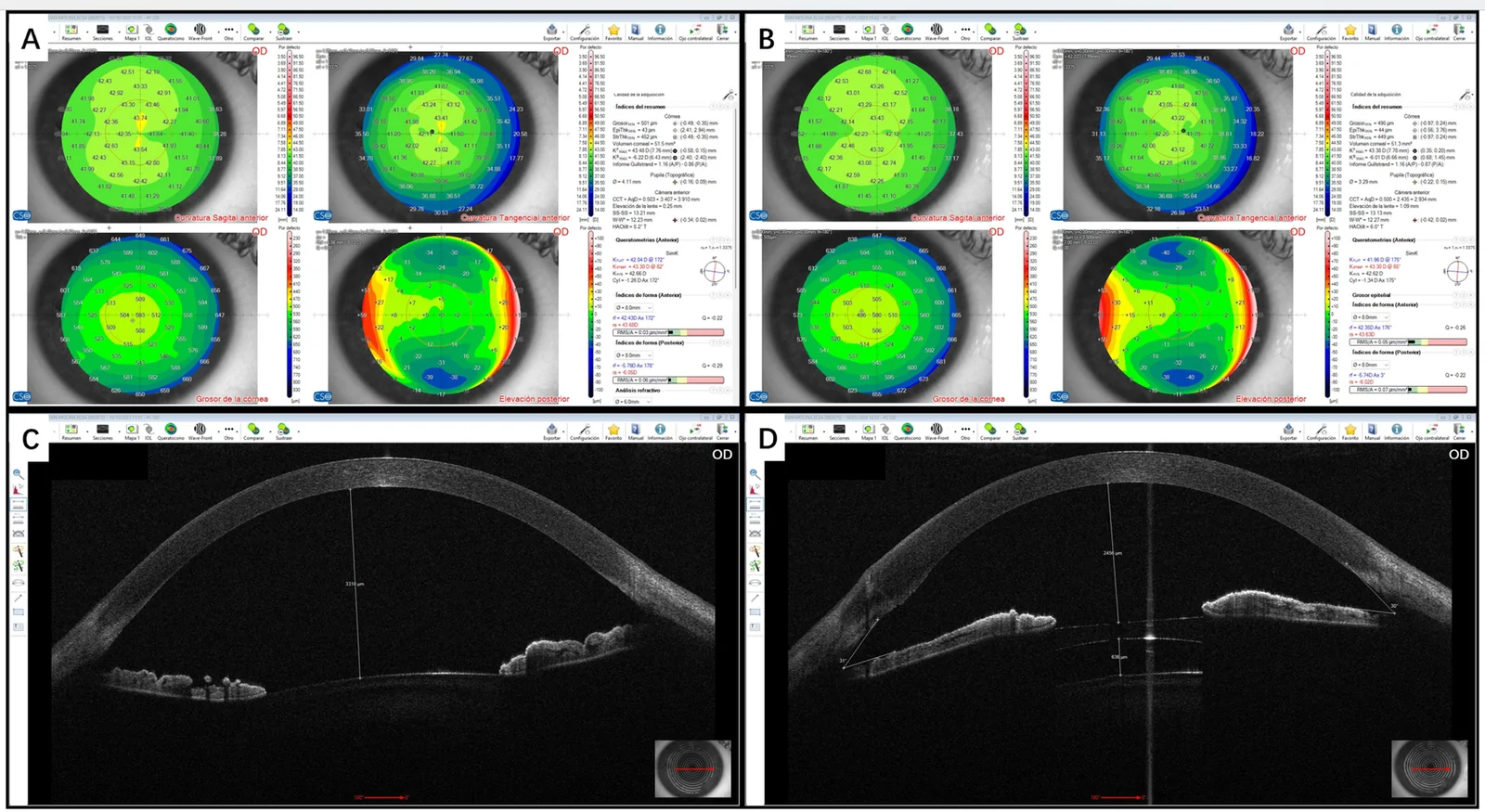
A 23-year-old female with stable keratoconus and high myopic astigmatism presented with severe visual impairment (preoperative UDVA: counting fingers at 1 m; CDVA: 1.0 with a manifest refraction of −10.00/−3.00 × 170°). At 2 years postoperatively, UDVA improved to 0.98 with a residual refraction of 0.00/−0.25 × 115°, and CDVA further improved to 1.2. **(A)** Preoperative corneal topography confirmed stable keratoconus, and anterior chamber depth was sufficient for phakic IOL implantation. **(B)** Postoperative corneal topography demonstrated maintained corneal stability. **(C)** Preoperative anterior segment OCT showed the baseline corneal contour with regular/irregular astigmatism and adequate anterior chamber depth. **(D)** Postoperative OCT confirmed preservation of the corneal surface profile and a well-centered posterior chamber ICL with an adequate vault.

**Figure 5:**
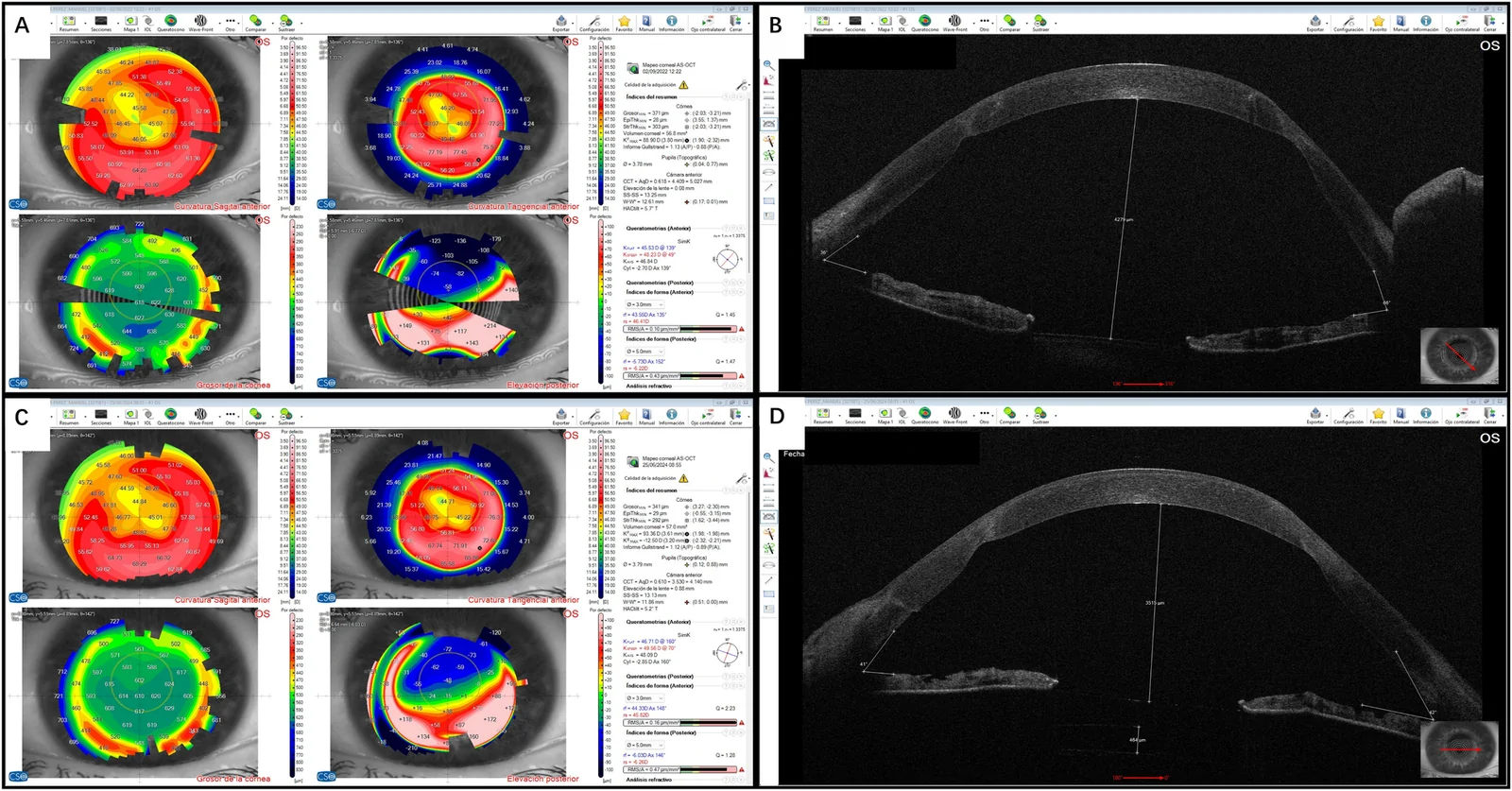
A 38-year-old male with a clear 7 mm corneal graft presented with poor visual quality due to high residual myopic astigmatism (preoperative UDVA: 0.05; CDVA: 0.80; manifest refraction: −5.50/−3.00 × 125°). At 1 month postoperatively, UDVA improved to 0.92 and CDVA to 0.96 with a small residual refraction of −0.25/−0.50 × 170°. At 12 months, UDVA was 0.86 and CDVA reached 1.0 with a refraction of 0.00/−1.00 × 180°, and at 19 months UDVA remained 0.80 with CDVA 1.0 and the same refraction, indicating long-term refractive stability. **(A)** Preoperative corneal topography and **(B)** anterior segment OCT after keratoplasty showing a stable graft–host interface, predominantly regular astigmatism, and an adequate anterior chamber configuration prior to toric EyeCryl phakic IOL implantation. **(C)** Postoperative corneal topography and **(D)** OCT demonstrating a stable corneal surface, a clear graft, and a well-centered toric EyeCryl phakic IOL with adequate vault.

Two major categories are currently used in KC: anterior chamber iris-fixated toric pIOLs (Figure 3) and posterior chamber toric pIOLs, most commonly toric implantable collamer lens (ICL) (Figure 4), with newer alternatives such as toric implantable phakic contact lens (IPCL) and EyeCryl toric (Figure 5) (74). Although both approaches have shown efficacy in selected stable eyes, posterior chamber lenses are currently more widely adopted in contemporary practice.

#### 4.4.2 Indications and limitations

The success of toric pIOL implantation in KC depends primarily on stability, regularity, refractive reproducibility and rotational stability. Candidates should have documented corneal stability, commonly after CXL, with no meaningful progression on serial tomography or refraction. The central cornea should be sufficiently clear, and visual limitation should not be dominated by dense stromal scarring or marked HOA-driven irregularity, because toric pIOLs cannot restore image quality in such eyes (74). Likewise, the refractive cylinder should be predominantly regular and reproducible. As a practical rule, when CDVA with RGP or scleral lenses is clearly superior to spectacle-corrected vision, the irregular component remains dominant and a toric pIOL alone is unlikely to provide optimal visual quality.

Reproducible manifest refraction and axis are therefore essential, ideally confirmed on repeated examinations and, when necessary, with a short spectacle trial before final lens selection. However, in toric pIOL implantation, reproducible refraction alone is insufficient; rotational stability is a major limiting factor, particularly in eyes with large cylinders. Even small postoperative rotations can induce clinically relevant residual astigmatism. Anatomical safety requirements must also be respected, including adequate anterior chamber depth and endothelial reserve for iris-fixated designs, and appropriate sizing and vault expectations for posterior chamber lenses (75, 76). These limitations underscore that the best candidates are eyes with stable KC, good CDVA, repeatable refraction, and no highly irregular astigmatism or marked HOAs (77). These selection criteria are particularly important because the main postoperative limitations of pIOLs in KC are closely related to anatomical safety and refractive stability. Iris-claw anterior chamber pIOLs avoid vault-related complications but require careful endothelial surveillance, whereas posterior chamber toric ICL series most commonly report transient early intraocular pressure (IOP) elevation and toric lens rotation, with repositioning required in approximately 5%–21% of eyes in larger KC series (12, 78–85). Although serious vision-threatening complications are uncommon in appropriately selected stable keratoconic eyes, endothelial cell loss, rotational instability, and persistent HOA-dominant visual limitation remain clinically relevant concerns requiring long-term follow-up (73, 81–102, 129).

#### 4.4.3 Results

In the recent review by Nowrouzi et al., which summarized the contemporary evidence on both iris-claw and posterior chamber pIOLs, satisfactory visual rehabilitation was consistently reported, with safety and efficacy indices generally close to or exceeding 1 (77). The strongest evidence currently exists for posterior chamber platforms. In a 2025 systematic review and meta-analysis including 16 observational studies and 397 eyes, ICL implantation produced a mean postoperative gain of 1.02 logMAR in UDVA, a reduction of 6.71 D in spherical equivalent, and a reduction of 2.06 D in cylinder, without significant adverse events (12). Likewise, in a 2025 cohort of posterior chamber pIOLs implanted in stable KC, UDVA improved from 0.76 ± 0.30 to 0.18 ± 0.17 logMAR, spherical equivalent from −3.6 ± 3.9 D to −0.07 ± 0.9 D, and refractive cylinder from −4.0 ± 2.2 D to −1.0 ± 1.1 D; predictability was good, with 67% of eyes within ± 0.50 D and 89% within ± 1.00 D of target spherical equivalent, and 53% and 74% within 0.50 D and 1.00 D of postoperative cylinder, respectively, although 4 eyes (18%) showed postoperative rotation and 2 required repositioning (103). Similar posterior chamber results were reported after CXL using toric IPCL, where sphere improved from −8.3 ± 3.6 D to −0.35 ± 0.2 D and cylinder from −3.4 ± 1.6 D to −0.53 ± 0.12 D at 12 months, with CDVA improving from 0.34 ± 0.13 to 0.18 ± 0.11 logMAR (104). More recently, EyeCryl Phakic Toric lenses also showed excellent refractive accuracy in 83 keratoconic eyes, with spherical equivalent improving from −8.19 ± 4.04 D to −0.06 ± 0.48 D, 77% of eyes within ± 0.50 D of target spherical equivalent, 92% with residual astigmatism ≤0.50 D, mean postoperative manifest astigmatism of only −0.28 ± 0.27 D, and no intraoperative or postoperative complications (105). Anterior chamber iris-fixated toric pIOLs also appear effective in selected ectatic eyes: in a 2024 series of 33 eyes, UDVA improved from 0.75 ± 0.35 to 0.02 ± 0.17 logMAR and CDVA from 0.16 ± 0.17 to 0.02 ± 0.17 logMAR, while vector analysis demonstrated moderate-to-good astigmatic predictability, with one-third of eyes within 0.50 D and one-half within 1.00 D of target refraction, a correction index of 1.08 ± 0.50, and an index of success of 0.44 ± 0.15; however, one eye required explantation because of spontaneous lens displacement (106). Long-term posterior chamber data also support durability, with 5-year follow-up showing spherical equivalent improvement from −5.35 ± 2.82 D to −0.78 ± 1.31 D and cylinder from −3.14 ± 1.58 D to −1.56 ± 1.53 D, while 82.5% of eyes achieved 6/12 or better UDVA and the safety and efficacy indices were 1.47 ± 0.32 and 1.24 ± 0.34, respectively (82). Taken together, phakic lenses should be regarded as the refractive finishing option that most directly addresses residual regular refractive astigmatism and myopia in appropriately selected eyes after corneal stabilization or prior corneal procedures, with posterior chamber toric platforms currently offering the strongest predictability data; however, their main limitation is that they do not regularize the cornea itself and are therefore less suitable when visual loss is dominated by severe irregular astigmatism or coma-related optical distortion (12). A more detailed discussion of the postoperative complications associated with this technique has been summarized elsewhere (12, 73, 77–79, 82, 86–91, 101, 102).

### 4.5 Cataract surgery and refractive lens exchange

**Figure 6:**
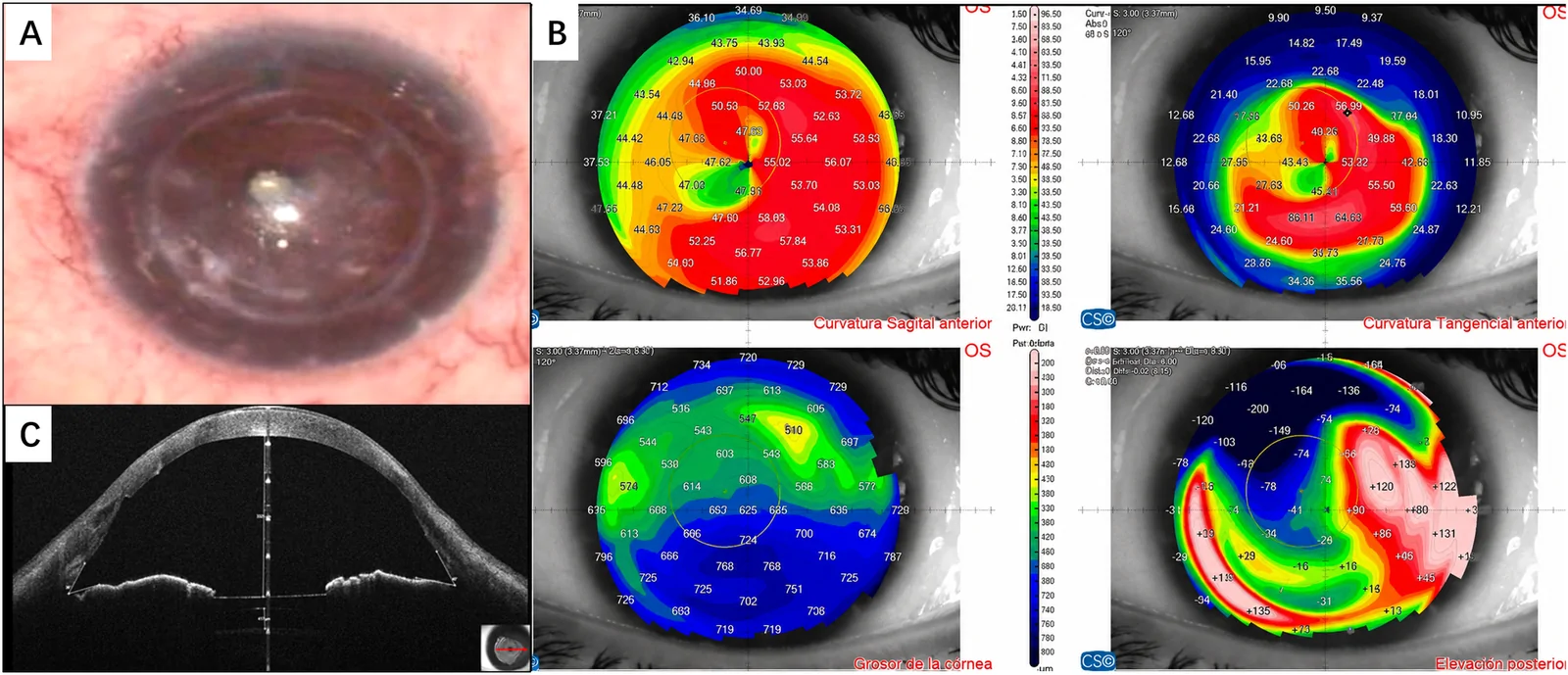
Management of extreme post-keratoplasty astigmatism during cataract surgery using a customized ultra–high-cylinder toric IOL. **(A)** A 40-year-old male with keratoconus previously treated with penetrating keratoplasty (left eye), and astigmatic keratotomy presented with visually significant cataract and severe residual myopic astigmatism (>12.0 D). **(B)** Corneal topography (MS-39) showing a steep cornea with irregular, asymmetric astigmatism. **(C)** Anterior segment OCT showing posterior chamber phakic refractive lens implantation. A bilensectomy procedure was performed (phakic lens explantation followed by phacoemulsification), and a customized hydrophobic foldable toric posterior chamber IOL (sphere −2.50 D; cylinder −12.75 D at 141°) was implanted to address the extreme corneal cylinder. Postoperatively, uncorrected distance visual acuity improved substantially, with a marked reduction in refractive cylinder (approximately −12.00 D to −3.75 D by 3 months), and the IOL remained well centered without clinically relevant rotation. The corneal graft remained clear, with no evidence of rejection or decompensation during follow-up.

Although this review focuses on therapeutic corneal refractive strategies for astigmatism in KC, cataract surgery merits a brief mention because it often becomes the decisive refractive step once lens opacity or dysfunctional lens changes contribute to visual limitation (Figure 6). Lens-based correction should be framed as a selected option when lens opacity or lens-related visual limitation is present: when the cornea is stable yet further corneal manipulation is unsafe or unlikely to improve optical quality, cataract extraction (or selected refractive lens exchange) can reduce ametropia and, in appropriate eyes, reduce astigmatism (107).

A key limitation is IOL power predictability in ectatic corneas, as conventional keratometric assumptions (including the anterior–posterior curvature relationship) are violated (108). Recent evidence supports KC-specific IOL formulas and tomography-informed corneal power estimation (including posterior corneal astigmatism or total keratometry). A recent network meta-analysis reported improved accuracy for KC-adapted approaches and recommended cross-checking multiple formulas, particularly as disease severity increases (109).

**Figure 7:**
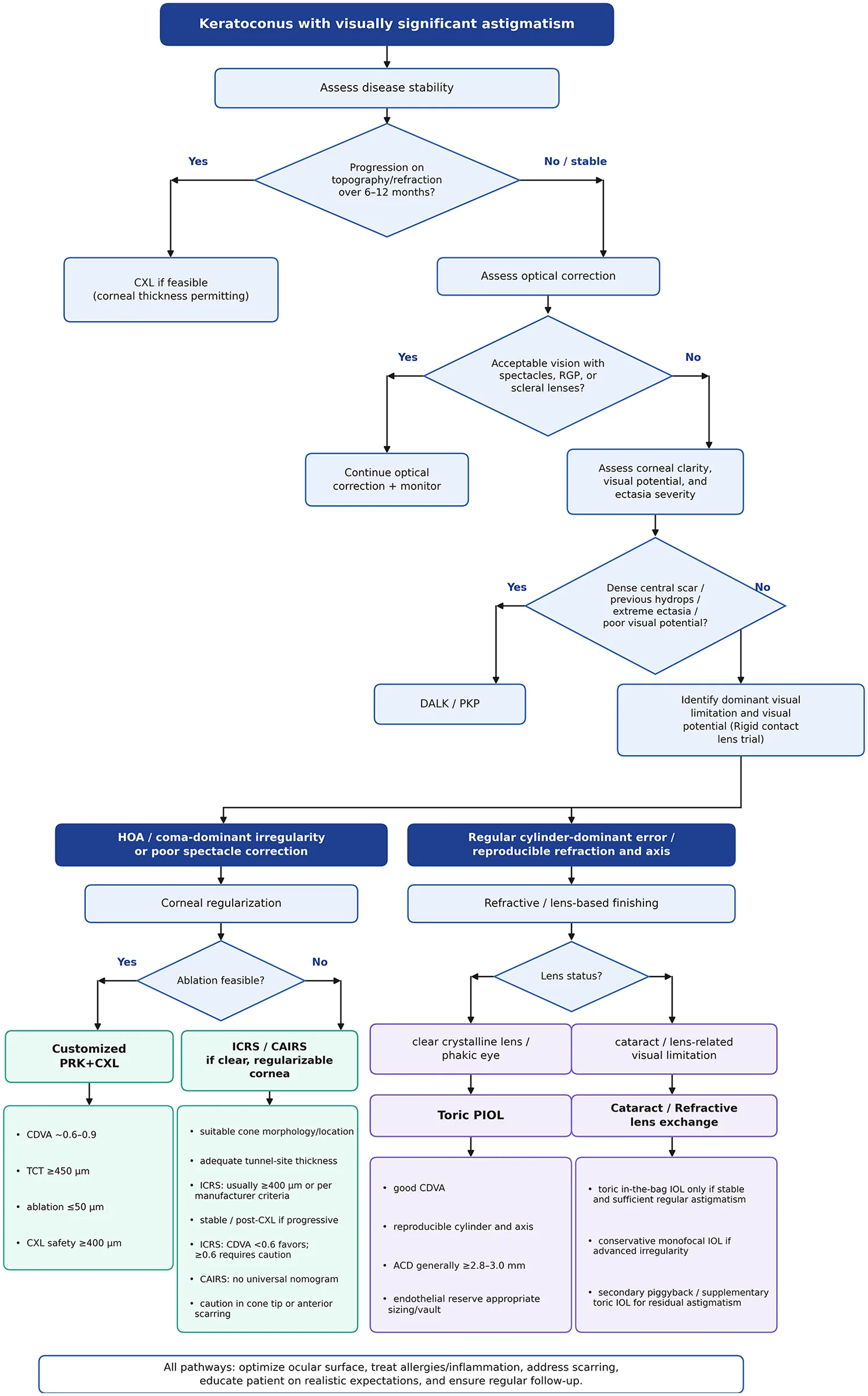
Individualized clinical decision framework for managing visually significant astigmatism in keratoconus. The branches represent alternative or complementary pathways selected according to disease activity, corneal phenotype, and the dominant source of visual limitation; patients do not necessarily proceed through all interventions. In particular, the feasibility of combined customized PRK + CXL should be determined by an integrated assessment of the planned ablation depth, residual stromal thickness, baseline corneal thickness, and CXL safety margin, with these practical criteria mainly based on (8). The indications and safety considerations for ICRS are mainly based on (10). ACD, anterior chamber depth; CAIRS, corneal allogeneic intrastromal ring segments; CDVA, corrected distance visual acuity; CXL, corneal collagen cross-linking; DALK, deep anterior lamellar keratoplasty; HOA, higher-order aberration; ICRS, intracorneal ring segments; IOL, intraocular lens; pIOL, phakic intraocular lens; PKP, penetrating keratoplasty; PRK, photorefractive keratectomy; RGP, rigid gas-permeable; TCT, thinnest corneal thickness.

For astigmatism management, toric IOLs can be effective when astigmatism is sufficiently regular and disease is mild–moderate with documented stability. A recent systematic review/meta-analysis of toric IOL implantation in KC (8 studies, 135 eyes) found consistent reductions in residual cylinder with meaningful improvements in UDVA and CDVA, supporting toric IOL use in carefully selected cases (110). In advanced KC or highly irregular astigmatism, toric predictability diminishes. These eyes are better managed with stabilization/regularization first (CXL ± ICRS, or keratoplasty where indicated), followed by a conservative lens-based strategy tailored to age. In older patients, toric in-the-bag IOL implantation may be more appropriate. In younger and middle-aged patients, a conservative monofocal IOL in the capsular bag followed by a secondary toric supplementary or piggyback IOL can avoid inducing additional, complex refractive issues and preserve future options. Finally, patients should be counseled that spectacles or contact lenses may still be required to optimize vision due to residual refractive error and/or HOAs (Figure 7 and Table 4).

**Table 4:** Practical comparison of major surgical approaches for keratoconus-related astigmatism.

Surgical approach	Surgical principle	Practical indications/selection criteria	Key limitations	Supporting references
Corneal collagen cross-linking (CXL)	Riboflavin saturation followed by ultraviolet-A irradiation to increase corneal biomechanical stability and halt ectatic progression.	Documented progression on tomography, keratometry, refraction, or clinical worsening, usually over 6–12 months; feasible when corneal thickness permits safe treatment.	Limited refractive predictability; does not directly neutralize astigmatism or HOAs; safety depends on corneal thickness and treatment protocol.	(3, 7–9)
Customized PRK + CXL	Tissue-sparing topography- or wavefront-guided surface ablation combined with simultaneous or sequential CXL to regularize the anterior corneal surface.	Mild-to-moderate KC with HOA/coma-dominant visual limitation and useful visual potential; typically CDVA ∼0.6–0.9, TCT ≥450 μm, planned ablation ≤50 μm, and post-ablation/CXL safety thickness ≥400 μm.	Requires adequate stromal reserve and high-quality topographic/wavefront data; refractive predictability is moderate; unsuitable for marked thinning, poor visual potential, or visually significant central scarring.	(1, 8, 15–29)
Intracorneal ring segments (ICRS)	Synthetic arc-shaped stromal implants inserted into intrastromal tunnels to flatten the cone, redistribute corneal stress, and reduce corneal asymmetry.	Contact lens intolerance, poor spectacle correction, or need for corneal regularization when ablation is not feasible; clear cornea; suitable cone morphology/location; tunnel-site thickness usually ≥400 μm or according to manufacturer criteria; CDVA <0.6 may favor indication, whereas CDVA ≥0.6 requires caution.	Nomogram-dependent; moderate refractive predictability; outcomes depend on cone morphology, segment selection, centration, tunnel depth, and baseline CDVA.	(10, 30–45)
Corneal allogeneic intrastromal ring segments (CAIRS)	Allogeneic corneal tissue segments implanted into stromal channels to provide biological corneal remodeling and stromal volume support.	Clear, regularizable KC; possible option in thinner corneas or when avoidance of synthetic material is preferred; may be considered when ICRS-like regularization is desired but biological stromal support is advantageous.	No universal nomogram; evidence remains less standardized than synthetic ICRS; long-term durability and optimal timing with CXL remain uncertain; caution is required in extremely ectatic corneas.	(11, 46–72)
Toric phakic intraocular lenses (pIOLs)	Anterior or posterior chamber toric phakic lens implantation to correct residual regular myopia and astigmatism without modifying corneal shape.	Stable or post-CXL KC; good CDVA; predominantly regular residual cylinder; reproducible manifest refraction and axis; adequate anterior segment anatomy; ACD generally ≥2.8–3.0 mm, with adequate endothelial reserve and appropriate sizing/vault.	Does not correct irregular astigmatism, coma, or corneal HOAs; requires stable refraction, reliable axis, adequate anatomy, and rotational stability.	(12, 73–106)
Cataract/lens-based surgery	Cataract extraction or refractive lens exchange with monofocal or toric IOL implantation; secondary piggyback toric correction may be considered in selected cases.	Cataract or lens-related visual limitation; stable KC; toric in-the-bag IOL only when astigmatism is sufficiently regular; conservative monofocal IOL preferred in advanced irregularity.	IOL power calculation is challenging; toric predictability decreases in advanced or highly irregular KC; residual spectacles, contact lenses, or secondary correction may still be needed.	(13, 107–110)

The practical indications and selection criteria summarized in this table integrate the cited evidence with expert clinical interpretation and should be individualized. Evidence levels and recommendation strengths are presented separately in Table 1.

ACD, anterior chamber depth; CAIRS, corneal allogeneic intrastromal ring segments; CDVA, corrected distance visual acuity; CXL, corneal collagen cross-linking; HOA, higher-order aberration; HOAs, higher-order aberrations; ICRS, intracorneal ring segments; IOL, intraocular lens; KC, keratoconus; pIOL, phakic intraocular lens; PRK, photorefractive keratectomy; TCT, thinnest corneal thickness.

## 5 Discussion and future directions

This review underscores that the surgical management of astigmatism in KC should not be conceptualized as simple cylindrical correction, but rather as the management of a complex optical disorder arising from the interaction of corneal asymmetry, irregular astigmatism, posterior corneal contribution, and coma-dominant HOAs. Accordingly, preoperative assessment is central to rational treatment selection.

Several principles emerge consistently from the literature. Astigmatism should be characterized using standardized and repeatable measurements that incorporate total corneal metrics, consistent centration and analysis zones, and confirmation of axis stability. Treatment selection should then be guided by the dominant clinical target—stabilization, corneal regularization, or residual refractive correction—together with stromal reserve, disease stability, epithelial masking, and visual potential.

The available procedures are complementary rather than interchangeable. Customized surface ablation and ICRS/CAIRS primarily address corneal irregularity, although their indications, tissue requirements, degree of standardization, and refractive predictability differ. Toric phakic IOLs and lens-based procedures are refractive finishing options for stable eyes with sufficiently regular residual astigmatism, but they do not address the underlying corneal irregularity.

This review has several limitations. First, as a structured narrative review based on a single-database search of English-language publications, it remains susceptible to selection, publication, and language bias and may not capture all relevant studies. Second, although the main clinical recommendations were graded using a predefined evidence hierarchy, no formal study-level risk-of-bias assessment or quantitative synthesis was performed. Third, the underlying evidence is heterogeneous in study design, patient selection, disease severity, surgical technique, outcome definitions, and follow-up duration, while direct head-to-head comparisons remain limited. These constraints are particularly relevant for emerging or less standardized interventions, including CAIRS and some lens-based strategies, for which the evidence is derived largely from observational studies with relatively short follow-up. Accordingly, the practical framework proposed in this review should be interpreted as a clinically oriented synthesis of the available evidence rather than a formal clinical guideline.

Future progress will likely depend on linking measurement, prediction, and reconstruction. Longitudinal multimodal imaging combined with interpretable artificial intelligence may help define KC phenotypes, predict progression patterns, and support more individualized treatment planning. In the long term, the goal should move beyond partial regularization toward safer and more precise shape restoration, potentially through rational combinations of additive stromal support and tissue-sparing regularization strategies.

## 6 Conclusion

1. Astigmatism in KC is a composite optical problem, not merely a manifest cylindrical refractive error.
2. Preoperative evaluation is decisive, and should include standardized assessment of total corneal astigmatism, centration, analysis zone, stromal reserve, disease stability, and measurement repeatability.
3. Treatment should follow an individualized, phenotype-driven logic: stabilize when progression is present, regularize when irregular corneal optics dominate visual impairment, and correct residual regular refractive error only after sufficient stability and optical regularity have been achieved; not every patient requires all three components.
4. Customized surface ablation and ICRS/CAIRS primarily act by corneal regularization, with improvement in visual quality deriving mainly from reduction of irregular astigmatism and HOA burden rather than exact cylinder neutralization.
5. Toric phakic IOLs are best considered refractive finishing procedures for stable eyes with sufficiently regular residual astigmatism.
6. No single procedure addresses all components of keratoconic astigmatism, so treatment must be individualized according to corneal phenotype and the dominant source of visual impairment.
